# Ethnic differences in metabolite signatures and type 2 diabetes: a nested case–control analysis among people of South Asian, African and European origin

**DOI:** 10.1038/s41387-017-0003-z

**Published:** 2017-12-19

**Authors:** Irene G. M. van Valkengoed, Carmen Argmann, Karen Ghauharali-van der Vlugt, Johannes M. F. G. Aerts, Lizzy M. Brewster, R. J. G. Peters, Frédéric M. Vaz, Riekelt H. Houtkooper

**Affiliations:** 1 Department of Public Health, Academic Medical Centre, I. van Valkengoed, Meibergdreef 9, J2-209, 1105 AZ Amsterdam, The Netherlands; 2 Department of Medical Biochemistry, Academic Medical Centre, I. van Valkengoed, Meibergdreef 9, J2-209, 1105 AZ Amsterdam, The Netherlands; 3 Department of Genetics and Genomic Sciences and Icahn Institute for Genomics and Multiscale Biology, Icahn School of Medicine at Mount Sinai, New York, NY USA; 4 Laboratory Genetic Metabolic Diseases, Academic Medical Centre, I. van Valkengoed, Meibergdreef 9, J2-209, 1105 AZ Amsterdam, The Netherlands; 5 Department of Biochemistry, Leiden Institute of Chemistry, Leiden, The Netherlands; 6 Department of Vascular Medicine, Academic Medical Centre, I. van Valkengoed, Meibergdreef 9, J2-209, 1105 AZ Amsterdam, The Netherlands; 7 Department of Cardiology, Academic Medical Centre, I. van Valkengoed, Meibergdreef 9, J2-209, 1105 AZ Amsterdam, The Netherlands

## Abstract

Accumulation of metabolites may mark or contribute to the development of type 2 diabetes mellitus (T2D), but there is a lack of data from ethnic groups at high risk. We examined sphingolipids, acylcarnitines and amino acids, and their association with T2D in a nested case–control study among 54 South Asian Surinamese, 54 African Surinamese and 44 Dutch in the Netherlands. Plasma metabolites were determined at baseline (2001–2003), and cumulative prevalence and incidence of T2D at follow-up (2011–2012). Weighted linear and logistic regression analyses were used to study associations. The mean level of most sphingolipids was lower, and amino-acid levels higher, in the Surinamese groups than among the Dutch. Surinamese individuals had higher mono- and polyunsaturated acylcarnitines and lower plasma levels of saturated acylcarnitine species than the Dutch. Several sphingolipids and amino acids were associated with T2D. Although only the shorter acylcarnitines seemed associated with prevalent T2D, we found an association of all acylcarnitines (except C0, C18 and C18:2) with incident T2D. Further analyses suggested a potentially different association of several metabolites across ethnic groups. Extension and confirmation of these findings may improve the understanding of ethnic differences and contribute to early detection of increased individual risk.

## Introduction

People of South Asian and African origin have a higher type 2 diabetes mellitus (T2D) prevalence and incidence compared with Europeans, at a given body mass index (BMI)^1–3^. Higher tissue and plasma concentrations of metabolites may contribute to the development of T2D. Prominent is the accumulation of lipids that culminates to lipotoxicity. Primary classes of intracellular lipid mediators associated with T2D and insulin resistance include cytosolic lipids such as sphingolipids, and mitochondria-derived metabolites such as acylcarnitines^4–7^. In addition to fat metabolites, there is a marked association between amino acids and T2D^8, 9^. Recent evidence suggests that associations may vary by ethnicity, for example, for amino acids in men^9^. This could signal differences in intake and the relative importance of metabolic processes across groups. However, data are limited. We examined ethnic differences in these metabolites and their association with T2D in people of South Asian, African and European origin in the Netherlands.

## Subjects and methods

### Study population

We carried out a nested case–control study. In brief, 1444 South Asian Surinamese, African Surinamese and European Dutch (henceforth Dutch) completed an interview and medical examination in the SUNSET study between 2001 and 2003^1, 3^. In 2011–2012, participants who still resided in Amsterdam were invited to take part in a follow-up examination within the HELIUS study^1, 2^.

We excluded individuals with missing data on T2D at either measurement or with self-reported baseline cardiovascular disease. From the group that remained, we took a sample from strata characterized by ethnicity, sex and T2D status at follow-up. Data for 54 South Asian Surinamese, 54 African Surinamese and 44 Dutch were available for analysis.

The Institutional Review Board of the Academic Medical Centre approved the studies. Participants provided a written informed consent.

### Measurements

Ethnicity was classified based on self-identification^1, 3^. BMI was calculated as weight in kilograms by height in meters squared. The covariate BMI change was calculated by subtracting BMI at follow-up from the baseline BMI. Healthy diet was defined in line with Nicolaou et al.^9^, based on 24 questions about the usual amount and frequency of intake of common food items and items more specific for the Surinamese (e.g., salty condiments). The individual dietary items were combined to form a composite score based on the Dutch guidelines for: fruit (2 points for two pieces of fruit or one piece of fruit and one glass of juice daily (no sugar added); 1 point for one piece of fruit daily or two pieces 5–6 days per week), vegetables (2 points for 200 g daily, 1 point for 150 g daily or 200 g 5–6 days per week), salt (1 point for minimal use of salt at cooking and at the table), fish (1 point for at least once a week), red meat (1 point for twice a week or less), breakfast (2 points for daily; 1 point for 5–6 days per week), fat in cooking (1 point for mostly oil).

Baseline T2D was defined as a fasting plasma glucose (HK/Glucose-6-P-dehydrogenase test; Roche-Diagnostics) ≥7.0 mmol/l and/or self-reported T2D. At follow-up, T2D was defined as an HbA1c ≥ 6.5% (48 mmol/mol) and/or a fasting plasma glucose ≥7.0 mmol/l and/or self-reported T2D^1, 10^.

In 2012, we measured metabolite concentrations in baseline plasma samples that had been stored at −80 °C. The analyses were done as reported previously^11, 12^.

### Statistical analysis

We used methods that weighted for the sampling strategy used. Baseline data were expressed as percentages or means with 95% confidence intervals. Ethnic differences in metabolites were calculated with linear regression, adjusted for the continuous variables age and BMI^1^; and, additionally, with adjustment for healthy diet score as a continuous variable. Sex was not considered, as it was previously not associated with T2D^3^ and had been used as sampling variable. Repeated analyses with non-parametric methods did not change results (data not shown). Subsequently, we examined the age and BMI adjusted association of the metabolites with cumulative T2D. In this analysis, we included all cases (*n* = 44), including those already identified at baseline. Moreover, we repeated the analysis with adjustment for BMI change and with further adjustment for healthy diet as a continuous variable. We also repeated the analysis after excluding persons with T2D at baseline, with additional adjustment for baseline fasting glucose. Finally, we evaluated differences in the association with cumulative T2D by ethnicity, reporting only report the metabolites with *p* ≤ 0.10 for the interaction.

Analyses were performed using the SAS package, version 9.3 (SAS Institute Inc., Cary, NC, USA). No power calculations were performed. We considered a *p* ≤ 0.05 indicative of an association (two sided).

## Results

Baseline characteristics differed by ethnicity (Supplement 1). The mean level of most sphingolipids was lower, and plasma amino-acid levels higher, in the Surinamese groups than the Dutch (Table 1; Supplement 2). For baseline acylcarnitine levels, there appears to be an overall switch in saturation, as Surinamese had higher mono- and polyunsaturated acylcarnitines and lower plasma levels of saturated acylcarnitine species. Additional adjustment for diet did not account for these differences (Supplement 3).

**Table 1:** The adjusted ethnic differences in sphingolipids, amino acids and acylcarnitines^a^

	South Asian Surinamese vs. Dutch			African Surinamese vs. Dutch			
	Mean difference	CI lower	CI upper	Mean difference	CI lower	CI upper	*p-*Value
Sphingolipids in µmol/l							
Cer d16:1	**−0.12**	**−0.20**	**−0.03**	**−0.15**	**−0.24**	**−0.05**	0.01
Cer d18:1	−0.48	−1.35	0.39	**−1.17**	**−2.06**	**−0.28**	0.01
Cer d18:2	0.10	−0.08	0.28	−0.08	−0.26	0.10	0.04
Gb3d18:1	−0.11	−0.22	0.01	0.07	−0.06	0.20	0.0001
Gb4 d18:1	**−0.27**	**−0.42**	**−0.13**	**−0.20**	**−0.37**	**−0.04**	0.001
HexCer d18:1	**−0.76**	**−1.31**	**−0.22**	−0.10	−0.69	0.48	<0.0001
HexCer/total Cer	**−0.06**	**−0.11**	**−0.01**	**0.06**	**0.01**	**0.12**	<0.0001
HexCer/cholesterol	**−0.11**	**−0.19**	**−0.04**	0.08	0.00	0.17	<0.0001
LacCer d18:1	−0.18	−0.51	0.14	−0.18	−0.40	0.43	0.25
Total Cer	−0.50	−1.59	0.59	**−1.39**	**−2.53**	**−0.26**	0.02
Total Cer/cholesterol	−0.05	−0.18	0.09	**−0.06**	**−0.21**	**0.09**	0.49
Amino acids in µmol/l							
Alanine	10.85	−16.40	38.10	**−50.16**	**−80.45**	**−19.87**	<0.0001
Arginine	**7.27**	**2.64**	**11.90**	**6.32**	**0.91**	**11.72**	0.008
Asparagine	**−19.09**	**−22.00**	**−16.18**	**−21.96**	**−25.29**	**−18.63**	<0.0001
Aspartic acid	**7.98**	**5.11**	**10.85**	3.03	−0.31	6.38	<0.0001
Citrulline	−0.24	−2.84	2.37	0.11	−3.55	3.77	0.97
Glutamine	−1.45	−42.67	39.76	−31.30	−78.16	15.56	0.28
Glutamic acid	**90.07**	**62.57**	**117.57**	**77.99**	**45.47**	**110.52**	<0.0001
Glycine	**53.73**	**29.00**	**78.46**	**66.86**	**36.51**	**97.22**	<0.0001
Isoleucine	**11.43**	**6.81**	**16.04**	**10.12**	**4.28**	**15.97**	<0.0001
Leucine	0.99	−6.92	8.89	−3.74	−12.90	5.43	0.39
Lysine	−4.97	−16.40	6.46	**−26.05**	**−38.62**	**−13.48**	<0.0001
Methionine	**5.29**	**3.77**	**6.80**	**4.14**	**2.21**	**6.06**	<0.0001
Ornithine	0.58	−5.59	6.69	**−10.29**	**−16.99**	**−3.58**	0.01
Phenylalanine	**5.34**	**2.09**	**8.60**	1.73	−2.07	5.53	0.003
Proline	**−63.86**	**−86.00**	**−41.72**	**−70.61**	**−95.84**	**−45.39**	<0.0001
Serine	5.18	−2.06	12.43	**9.92**	**1.59**	**18.26**	0.07
Tryptophan	−1.57	−4.44	1.30	**−3.52**	**−6.75**	**−0.29**	0.07
Tyrosine	**6.61**	**2.44**	**10.77**	**5.11**	**0.49**	**9.74**	0.009
Valine	**35.45**	**19.80**	**51.09**	**29.92**	**8.64**	**51.20**	<0.0001
Acylcarnitines in µmol/l							
C0	−0.20	−2.18	1.79	−1.80	−4.05	0.46	0.17
C2	−0.14	−0.69	0.40	−0.50	−1.09	0.08	0.19
C3 (*10^−2^)	2.88	−0.97	6.72	1.41	−3.70	6.52	0.32
C4 (*10^−2^)	−1.06	−5.75	3.64	**−5.65**	**−10.63**	**−0.68**	0.001
C5 (*10^−2^)	**−1.35**	**−2.50**	**−0.20**	**−1.54**	**−2.84**	**−0.24**	0.05
C6 (*10^−2^)	0.39	−0.52	1.31	1.04	−0.24	2.31	0.28
C8 (*10^−2^)	−1.24	−3.96	1.47	1.99	−2.42	6.41	0.23
C10	**−0.05**	**−0.09**	**−0.01**	0.01	−0.06	0.07	0.01
C12 (*10^−2^)	−1.00	−2.07	0.06	−0.69	−2.22	0.84	0.18
C14 (*10^−2^)	**−0.97**	**−1.39**	**−0.55**	**−0.91**	**−1.43**	**−0.39**	<0.0001
C16 (*10^−2^)	**−1.80**	**−2.56**	**−1.04**	**−1.98**	**−2.88**	**−1.09**	<0.0001
C18 (*10^-2^)	**−1.19**	**−1.56**	**−0.82**	**−1.04**	**−1.45**	**−0.63**	<0.0001
C10:1 (*10^-2^)	**9.09**	**6.07**	**12.11**	**7.36**	**2.76**	**11.96**	<0.0001
C12:1 (*10^−2^)	−1.33	−2.73	0.06	−0.80	−2.99	1.39	0.15
C14:1 (*10^−2^)	−1.11	−2.69	0.48	−0.71	−3.03	1.60	0.38
C14:2 (*10^−2^)	**3.09**	**1.97**	**4.21**	**1.77**	**0.32**	**3.21**	<0.0001
C16:1 (*10^−2^)	**−0.57**	**−0.97**	**−0.18**	**−0.65**	**−1.16**	**−0.14**	0.01
C18:1 (*10^−2^)	**−1.88**	**−2.92**	**−0.85**	**−1.58**	**−2.91**	**−0.25**	0.002
C18:2 (*10^−2^)	**3.67**	**2.89**	**4.44**	**1.79**	**0.79**	**2.78**	<0.0001

^a^Adjusted for age and baseline body mass index; CI = 95% confidence interval; *p*-value = *p*-value for the adjusted comparison between ethnic groups (F-test). Please note that no corrections for multiple testing were applied. Bold marking indicates values that are significantly different from the European Dutch

Metabolite levels were associated with prevalent T2D (Table 2). Adjustment for diet or BMI change did not affect our interpretation (Supplement 5). The restricted analyses (incident only) also largely confirmed the associations for sphingolipids and amino acids; a limited number of metabolites, for example, alanine and leucine, were no longer associated, whereas others appeared similarly or even more strongly associated (e.g., lactosylceramide, methionine and proline; Table 2). For the acylcarnitines, however, the difference was notable. Although only the shorter acylcarnitines seemed associated with cumulative T2D, we found an association of higher mean baseline levels of all acylcarnitines (except C0, C18 and C18:2) with incident T2D. Further analyses showed some evidence for a differential association of several metabolites with the T2D risk by ethnicity (Supplement 5).

**Table 2:** The adjusted association of sphingolipids, amino acids and AC with type 2 diabetes

	Cumulative prevalence type 2 diabetes^a^				Incident type 2 diabetes only^b^			
	OR	CI lower	CI upper	*p-*Value	OR	CI lower	CI upper	*p-*Value
Sphingolipids								
Cer d16:1	2.02	1.42	2.89	<0.0001	1.46	0.77	2.77	0.24
Cer d18:1	2.36	1.51	3.69	0.0001	1.93	1.11	3.36	0.02
Cer d18:2	1.81	1.24	2.63	0.0019	1.21	0.72	2.01	0.47
Gb3d18:1	0.66	0.44	1.00	0.05	0.63	0.37	1.07	0.09
Gb4 d18:1	0.99	0.69	1.42	0.95	0.75	0.45	1.26	0.27
HexCer d18:1	1.03	0.73	1.45	0.87	1.01	0.53	1.94	0.97
HexCer/total Cer	0.36	0.23	0.58	<0.0001	0.49	0.26	0.90	0.02
HexCer/cholesterol	0.80	0.57	1.13	0.21	1.34	0.77	2.32	0.29
LacCer d18:1	1.34	0.90	2.00	0.14	2.15	1.27	3.61	0.004
Total Cer	2.35	1.54	3.59	<0.0001	1.85	1.03	3.32	0.04
Total Cer/cholesterol	2.79	1.95	3.98	<0.0001	3.22	1.58	6.53	0.001
Amino acids								
Alanine	1.44	1.01	2.07	0.045	1.01	0.54	1.89	0.96
Arginine	1.23	0.91	1.67	0.17	1.15	0.65	2.05	0.63
Asparagine	0.37	0.20	0.67	0.001	0.11	0.02	0.64	0.01
Aspartic acid	1.28	0.94	1.74	0.12	1.49	1.00	2.23	0.05
Citrulline	1.05	0.80	1.39	0.72	1.09	0.65	1.83	0.73
Glutamine	0.43	0.30	0.62	<0.0001	0.34	0.19	0.63	0.0005
Glutamic acid	0.84	0.60	1.18	0.31	1.35	0.86	2.12	0.19
Glycine	0.87	0.59	1.28	0.47	1.15	0.72	1.85	0.56
Isoleucine	2.62	1.53	4.47	0.0004	1.80	1.02	3.18	0.04
Leucine	2.10	1.42	3.10	0.0002	1.36	0.74	2.50	0.32
Lysine	1.20	0.83	1.74	0.32	0.84	0.45	1.57	0.58
Methionine	1.30	0.96	1.76	0.09	1.71	1.11	2.65	0.01
Ornithine	0.80	0.58	1.10	0.16	0.56	0.27	1.13	0.10
Phenylalanine	1.32	0.96	1.81	0.08	1.36	0.80	2.31	0.25
Proline	0.88	0.59	1.32	0.54	0.33	0.13	0.83	0.02
Serine	0.70	0.49	0.99	0.04	0.81	0.49	1.36	0.43
Tryptophan	1.13	0.87	1.48	0.36	0.87	0.40	1.50	0.44
Tyrosine	1.25	0.85	1.85	0.25	0.83	0.43	1.60	0.58
Valine	2.03	1.27	3.25	0.003	1.79	1.15	2.78	0.009
Acylcarnitines								
C0	1.28	0.98	1.67	0.07	1.37	0.85	2.20	0.19
C2	1.26	0.93	1.71	0.13	1.89	1.04	3.42	0.04
C3	1.33	1.02	1.75	0.04	1.55	1.04	2.29	0.03
C4	1.85	1.36	1.52	<0.0001	1.88	1.23	1.86	0.003
C5	1.76	1.25	2.47	0.001	2.10	1.17	3.77	0.01
C6	1.44	1.11	1.86	0.005	1.77	1.21	1.59	0.003
C8	1.33	1.06	1.67	0.01	1.65	1.20	2.27	0.002
C10	1.26	0.98	1.62	0.07	1.58	1.15	2.17	0.005
C12	1.24	0.93	1.66	0.14	1.80	1.17	2.78	0.007
C14	1.36	0.99	1.86	0.06	2.39	1.41	4.04	0.001
C16	1.01	0.70	1.46	0.97	1.65	0.93	2.93	0.09
C18	0.81	0.58	1.13	0.21	0.97	0.54	1.75	0.93
C10:1	1.40	1.10	1.77	0.007	1.68	1.19	2.37	0.003
C12:1	1.06	0.82	1.38	0.66	1.50	0.98	2.29	0.06
C14:1	1.16	0.88	1.53	0.30	1.77	1.14	2.75	0.01
C14:2	1.17	0.88	1.55	0.27	1.76	1.15	2.68	0.008
C16:1	1.02	0.78	1.34	0.88	1.48	0.99	2.22	0.05
C18:1	0.97	0.71	1.32	0.83	1.63	1.10	2.40	0.01
C18:2	1.09	0.84	1.43	0.51	1.31	0.77	2.26	0.32

^a^Adjusted for age, ethnicity, baseline body mass index

^b^Adjusted for age, ethnicity, baseline body mass index, baseline fasting glucose. People with prevalent type 2 diabetes at baseline were excluded. OR = odds ratio per standard deviation increase; CI = 95 % confidence interval; *p*-value = *p*-value for the adjusted association with type 2 diabetes (likelihood ratio test). Please note that no corrections for multiple testing were applied

## Discussion

South Asian and African Surinamese had lower sphingolipid, higher plasma amino acid, higher mono- and polyunsaturated acylcarnitine and lower saturated acylcarnitine plasma levels than the Dutch. Differences in these metabolites were associated with cumulative and incident T2D. The observed differences in metabolite levels may reflect the specific dietary intakes of the ethnic groups in the Netherlands^2, 13^. However, the available self-reported data on diet did not explain associations in the current study. In addition, differences may reflect ethnic differences in (nutrient) metabolism. A difference in brown adipose tissue volume and capacity in South Asians has been found that may contribute to an reduced energy expenditure and T2D risk^14^.

We found that higher levels of several sphingolipids, for example, ceramide and lactosylceramide, were associated with T2D, with evidence for a somewhat stronger association among those of African origin than others. To the best of our knowledge, no data have been reported for Africans or South Asians. However, the association of ceramide is consistent with the reported association in Australians and Mexican Americans^6^.

The association of acylcarnitines with T2D also fits reported associations of several, in particular short and medium chain, acylcarnitines with prevalent T2D in obese African-American women^5^. However, another study did not find strong associations with insulin resistance^4^. Differences in the association between African, South Asian and European origin populations were not reported.

The associations of T2D with the three branched-chain amino acids (BCAAs) and to a lesser extent phenylalanine, as well as the lack of association with, for example, ornithine, citrulline and arginine, are mostly in line with previous work^4, 7^. However, a recent study did not find an association of BCAA in South Asian family members of people with T2D^15^. The association of tyrosine was consistent with one study, and inconsistent with another^4, 7^. Finally, our study suggested some associations with other amino acids, for example, the negative association with serine and proline, which were not found previously^4^.

Ethnic differences in associations with incident T2D, for instance for BCAA and tyrosine, have been reported^7^. Interestingly, these differences were not significant in our study, but we found some evidence for a possibly different association of other amino acids with cumulative T2D. Yet, the numbers are too small to draw firm conclusions.

If confirmed, our findings may have implications for the use of metabolites to enhance risk assessment for incident diabetes^16^. Further exploration of ethnic differences may also point toward new pathways underlying the excess burden of T2D in some groups.

### Limitations

Our observational data have several limitations. First, causality cannot be inferred. Second, we determined metabolite levels in stored plasma samples, which may better reflect levels in certain tissues and organs than others^17^. Reported levels may not be reflective of all (changes in) relevant metabolic processes.

Third, our analysis was based on cumulative cases at follow-up. Results of the analysis of incident cases largely confirmed the associations, but the power was limited and we could not explore all potential confounders and interactions.

Finally, in line with previous work^9^, we chose not to apply a correction for multiple testing. Although this may imply some false-positive results, even a strict Bonferroni correction would not have explained all associations.

In conclusion, we found large ethnic differences in sphingolipids, acylcarnitines and amino acids, which were associated with T2D in people of South Asian, African and European origin. Extension and confirmation of these findings may improve the understanding of ethnic differences in T2D and contribute to early detection of increased individual risk.

## Electronic supplementary material

Supplement 1

Supplement 2

Supplement 3

Supplement 4

Supplement 5

## References

[1] Admiraal WM (2014). Ethnic disparities in the association of impaired fasting glucose with the 10-year cumulative incidence of type 2 diabetes. Diabetes Res. Clin. Pract..

[2] Dekker LH (2015). Comparable dietary patterns describe dietary behavior across ethnic groups in the Netherlands, but different elements in the diet are associated with glycated hemoglobin and fasting glucose concentrations. J. Nutr..

[3] Bindraban NR (2008). Prevalence of diabetes mellitus and the performance of a risk score among Hindustani Surinamese, African Surinamese and ethnic Dutch: a cross-sectional population-based study. BMC Public Health.

[4] Palmer ND (2015). Metabolomic profile associated with insulin resistance and conversion to diabetes in the Insulin Resistance Atherosclerosis Study. J. Clin. Endocrinol. Metab..

[5] Adams SH (2009). Plasma acylcarnitine profiles suggest incomplete long-chain fatty acid beta-oxidation and altered tricarboxylic acid cycle activity in type 2 diabetic African-American women. J. Nutr..

[6] Meikle PJ (2013). Plasma lipid profiling shows similar associations with prediabetes and type 2 diabetes. PLoS ONE.

[7] Holland WL, Summers SA (2008). Sphingolipids, insulin resistance, and metabolic disease: new insights from in vivo manipulation of sphingolipid metabolism. Endocr. Rev..

[8] Wang TJ (2011). Metabolite profiles and the risk of developing diabetes. Nat. Med..

[9] Tillin T (2015). Diabetes risk and amino acid profiles: cross-sectional and prospective analyses of ethnicity, amino acids and diabetes in a South Asian and European cohort from the SABRE (Southall And Brent REvisited) Study. Diabetologia.

[10] American Diabetes Association. (2015). Classification and diagnosis of diabetes. Diabetes Care.

[11] Groener JE (2007). HPLC for simultaneous quantification of total ceramide, glucosylceramide, and ceramide trihexoside concentrations in plasma. Clin. Chem..

[12] Houtkooper RH (2011). The metabolic footprint of aging in mice. Sci. Rep..

[13] Nicolaou M, van Dam RM, Stronks K (2006). Acculturation and education level in relation to quality of the diet: a study of Surinamese South Asian and Afro-Caribbean residents of the Netherlands. J. Hum. Nutr. Diet..

[14] Bakker LE (2014). Brown adipose tissue volume in healthy lean south Asian adults compared with white Caucasians: a prospective, case-controlled observational study. Lancet Diabetes Endocrinol..

[15] Jainandunsing S (2016). Discriminative ability of plasma branched-chain amino acid levels for glucose intolerance in families at risk for type 2 diabetes. Metab. Syndr. Relat. Disord..

[16] Sun L (2016). Early prediction of developing type 2 diabetes by plasma acylcarnitines: a population-based study. Diabetes Care.

[17] Schooneman MG (2015). Transorgan fluxes in a porcine model reveal a central role for liver in acylcarnitine metabolism. Am. J. Physiol. Endocrinol. Metab..

