# Supplementary material for: Ethnic differences in metabolite signatures and type 2 diabetes: a nested case–control analysis among people of South Asian, African and European origin
Source: Nutr Diabetes. 2017 Dec 19;7(12):300. doi: 10.1038/s41387-017-0003-z (PMC5865542; doi:10.1038/s41387-017-0003-z)
Supplement: Supplementary file 1 — Supplement 1 [file 41387_2017_3_MOESM1_ESM.docx]

**Supplement 1. Characteristics of participants in the study**

|  | **South Asian**  **Surinamese (n=54)** | | | **African**  **Surinamese (n=54)** | | | **European Dutch**  **(n=44)** | | |
| --- | --- | --- | --- | --- | --- | --- | --- | --- | --- |
|  | Mean or % | CI lower | CI upper | Mean or % | CI lower | CI upper | Mean or % | CI lower | CI upper |
| **Mean age at baseline (years)** | 45.3 | 44.0 | 46.6 | 44.6 | 43.1 | 46.2 | 46.9 | 45.1 | 48.8 |
| **Sex (% men) ^a^** | 46.2 |  |  | 34.8 |  |  | 47.7 |  |  |
| **Mean BMI at baseline (kg/m2)** | **26.3** | **25.7** | **27.0** | **28.4** | **26.5** | **30.3** | **25.4** | **24.5** | **26.4** |
| **Mean waist circumference at baseline (cm)** | 92.5 | 90.7 | 94.4 | 94.5 | 89.8 | 99.2 | 89.1 | 86.1 | 92.0 |
| **Mean cholesterol at baseline (mmol/l)** | **5.5** | **5.3** | **5.7** | **5.0** | **4.8** | **5.3** | **5.8** | **5.5** | **6.0** |
| **Mean HDL-cholesterol at baseline (mmol/l)** | **1.3** | **1.2** | **1.3** | **1.5** | **1.4** | **1.6** | **1.5** | **1.5** | **1.6** |
| **Mean triglycerides at baseline (mmol/l)** | **1.4** | **1.2** | **1.5** | **0.89** | **0.79** | **0.98** | **1.2** | **1.0** | **1.4** |
| **Hypertension at baseline (%)^b^** | 30.6 |  |  | 31.5 |  |  | 14.1 |  |  |
| **- Systolic blood pressure (mmHg)** | **125.8** | **122.6** | **129.0** | **126.3** | **121.6** | **131.0** | **118.3** | **113.2** | **123.4** |
| **- Diastolic blood pressure (mmHg)** | **83.3** | **81.1** | **85.5** | **82.0** | **79.5** | **84.5** | **75.7** | **72.6** | **78.8** |
| **Diabetes at baseline (%)^a,c^** | 19.5 |  |  | 9.8 |  |  | 2.8 |  |  |
| **- Mean baseline FPG (mmol/l)** | **6.0** | **5.8** | **6.2** | **5.8** | **5.6** | **6.0** | **5.3** | **5.1** | **5.4** |

CI= 95%-confidence interval; bold indicates a Chi-square or Anova test p<0.05 for the difference between ethnic groups (sampling variables were excluded from comparisons); **^a^** (Part of sampling variable; **^b^** Definition: systolic blood pressure (SBP) ≥ 140 mmHg, or diastolic blood pressure (DBP) ≥ 90 mmHg, or being on antihypertensive therapy; **^c^** Defined as an FPG ≥ 7.0 mmol/L and/or self-reported type 2 diabetes, excluding people who only reported gestational diabetes.
