# Supplementary material for: Ethnic differences in metabolite signatures and type 2 diabetes: a nested case–control analysis among people of South Asian, African and European origin
Source: Nutr Diabetes. 2017 Dec 19;7(12):300. doi: 10.1038/s41387-017-0003-z (PMC5865542; doi:10.1038/s41387-017-0003-z)
Supplement: Supplementary file 2 — Supplement 2 [file 41387_2017_3_MOESM2_ESM.doc]

**Supplement 2**. **Mean levels of metabolites by ethnic group**

|  | **South AsianSurinamese**  **(n=54)** | | **African Surinamese**  **(n=54)** | | **Dutch**  **(n=44)** | |
| --- | --- | --- | --- | --- | --- | --- |
|  | ***Mean*** | ***SEM*** | ***Mean*** | ***SEM*** | ***Mean*** | ***SEM*** |
| *Sphingolipids in µmol/L* |  |  |  |  |  |  |
| **Cer d16:1** | **0.60** | **0.02** | **0.56** | **0.02** | **0.73** | **0.04** |
| **Cer d18:1** | **7.73** | **0.19** | **7.02** | **0.17** | **8.28** | **0.37** |
| **Cer d18:2** | **1.51** | **0.05** | **1.33** | **0.04** | **1.43** | **0.07** |
| **Gb3 d18:1** | **1.10** | **0.02** | **1.27** | **0.04** | **1.21** | **0.05** |
| **Gb4 d18:1** | **0.73** | **0.03** | **0.80** | **0.04** | **1.01** | **0.06** |
| **HexCer d18:1** | **2.23** | **0.05** | **2.41** | **0.14** | **2.41** | **0.14** |
| **HexCer/Total Cer** | **0.41** | **0.01** | **0.54** | **0.02** | **0.46** | **0.02** |
| **HexCer/Cholesterol** | **0.71** | **0.02** | **0.91** | **0.03** | **0.81** | **0.03** |
| **LacCer d18:1** | 0.52 | 0.01 | 0.68 | 0.02 | 0.58 | 0.03 |
| **Total Cer** | **3.92** | **0.09** | **4.61** | **0.12** | **4.66** | **0.24** |
| **Total Cer/Cholesterol** | 1.76 | 0.03 | 1.75 | 0.04 | 1.81 | 0.06 |
| *Aminoacids in µmol/L* |  |  |  |  |  |  |
| **Alanine** | **355.25** | **6.27** | **295.90** | **10.85** | **347.18** | **12.61** |
| **Arginine** | **59.58** | **1.25** | **57.99** | **2.01** | **52.73** | **1.97** |
| **Asparagine** | **28.68** | **0.77** | **25.86** | **1.31** | **47.78** | **1.18** |
| **Aspartic acid** | **25.34** | **1.19** | **20.43** | **1.50** | **17.33** | **0.71** |
| **Citrulline** | 33.04 | 0.70 | 33.00 | 1.50 | 33.93 | 1.15 |
| **Glutamine** | 625.35 | 12.42 | 598.91 | 19.26 | 630.74 | 17.47 |
| **Glutamic acid** | **484.23** | **8.46** | **471.49** | **13.67** | **397.41** | **10.36** |
| **Glycine** | **230.45** | **8.67** | **241.53** | **11.98** | **177.23** | **8.70** |
| **Isoleucine** | **75.98** | **1.06** | **75.11** | **2.27** | **64.79** | **1.96** |
| **Leucine** | 133.87 | 1.75 | 130.20 | 3.17 | 133.04 | 3.44 |
| **Lysine** | **176.28** | **2.55** | **157.74** | **4.33** | **180.18** | **5.04** |
| **Methionine** | **28.25** | **0.51** | **27.07** | **0.77** | **22.95** | **0.52** |
| **Ornithine** | **72.94** | **1.88** | **62.14** | **2.81** | **73.34** | **2.63** |
| **Phenylalanine** | **65.77** | **0.87** | **62.69** | **1.40** | **60.51** | **1.37** |
| **Proline** | **172.30** | **3.98** | **165.21** | **7.64** | **238.82** | **10.49** |
| **Serine** | **95.44** | **1.92** | **100.54** | **2.83** | **89.82** | **2.99** |
| **Tryptophan** | **59.36** | **0.65** | **56.65** | **0.86** | **61.73** | **1.31** |
| **Tyrosine** | **60.81** | **1.19** | **60.11** | **1.87** | **54.30** | **1.73** |
| **Valine** | **264.55** | **4.49** | **259.59** | **8.32** | **229.79** | **6.31** |
| *Acylcarnitines in µmol/L* |  |  |  |  |  |  |
| **C0** | 35.21 | 0.50 | 33.83 | 0.81 | 35.42 | 0.89 |
| **C2** | 5.11 | 0.17 | 4.79 | 0.16 | 5.27 | 0.20 |
| **C3 (*10^-2^)** | 38.27 | 1.03 | 36.88 | 1.84 | 35.53 | 1.53 |
| **C4 (*10^-2^)** | **25.88** | **0. 78** | **21.56** | **0. 92** | **26.73** | **2.03** |
| **C5 (*10^-2^)** | **8.80** | **0.22** | **8.63** | **0.38** | **10.23** | **0.50** |
| **C6 (*10^-2^)** | 6.43 | 0.27 | 7.12 | 0.49 | 6.12 | 0.34 |
| **C8 (*10^-2^)** | 15.33 | 0.80 | 18.46 | 1.75 | 16.89 | 0.97 |
| **C10** | **0.20** | **0.01** | **0.26** | **0.03** | **0.26** | **0.02** |
| **C12 (*10^-2^)** | 6.62 | 0.30 | 6.88 | 0.51 | 7.75 | 0.39 |
| **C14 (*10^-2^)** | **2.74** | **0.11** | **2.75** | **0.15** | **3.78** | **0.17** |
| **C16 (*10^-2^)** | **8.28** | **0.21** | **8.10** | **0.26** | **10.20** | **0.35** |
| **C18 (*10-2)** | **2.50** | **0.07** | **2.64** | **0. 10** | **3.73** | **0.18** |
| **C10:1 (*10-2)** | **24.21** | **1.10** | **22.44** | **1.92** | **15.39** | **0.86** |
| **C12:1 (*10^-2^)** | 6.97 | 0.38 | 7.44 | 0.71 | 8.45 | 0.47 |
| **C14:1 (*10^-2^)** | 7.77 | 0.47 | 8.00 | 0.75 | 9.13 | 0.55 |
| **C14:2 (*10^-2^)** | **7.56** | **0.44** | **6.12** | **0.48** | **4.65** | **0.26** |
| **C16:1 (*10^-2^)** | **2.17** | **0.12** | **2.09** | **0.15** | **2.79** | **0.15** |
| **C18:1 (*10^-2^)** | **8.89** | **0.27** | **9.20** | **0.35** | **10.90** | **0.43** |
| **C18:2 (*10^-2^)** | **8.54** | **0.29** | **6.59** | **0.32** | **5.02** | **0.24** |

SEM= standard error of the mean; Bold indicates an p<0.05 for the univariate difference between the ethnic groups (Anova test).
