# Supplementary material for: Ethnic differences in metabolite signatures and type 2 diabetes: a nested case–control analysis among people of South Asian, African and European origin
Source: Nutr Diabetes. 2017 Dec 19;7(12):300. doi: 10.1038/s41387-017-0003-z (PMC5865542; doi:10.1038/s41387-017-0003-z)
Supplement: Supplementary file 3 — Supplement 3 [file 41387_2017_3_MOESM3_ESM.docx]

**Supplement 3. The adjusted ethnic differences in sphingolipids, amino acids and acylcarnitines, with further adjustment for diet**^a^

|  | South Asian Surinamese vs. Dutch |  |  | African Surinamese  vs. Dutch |  |  |  |
| --- | --- | --- | --- | --- | --- | --- | --- |
|  | **Mean difference** | **CI**  **lower** | **CI**  **upper** | **Mean difference** | **CI**  **lower** | **CI**  **upper** | ***P value*** |
| *Sphingolipids in µmol/L* |  |  |  |  |  |  |  |
| **Cer d16:1** | **-0.12** | **-0.21** | **-0.03** | **-0.14** | **-0.24** | **-0.05** | *0.01* |
| **Cer d18:1** | -0.48 | -1.29 | 0.34 | **-1.17** | **-2.10** | **-0.24** | *0.02* |
| **Cer d18:2** | 0.09 | -0.08 | 0.26 | -0.07 | -0.26 | 0.12 | *0.06* |
| **Gb3d18:1** | -0.10 | -0.22 | 0.01 | 0.07 | -0.06 | 0.20 | *0.0002* |
| **Gb4 d18:1** | **-0.29** | **-0.43** | **-0.15** | **-0.19** | **-0.35** | **-0.03** | *0.0002* |
| **HexCer d18:1** | **-0.77** | **-1.30** | **-0.23** | -0.10 | -0.70 | 0.50 | *<0.0001* |
| **HexCer/Total Cer** | **-0.06** | **-0.11** | **-0.01** | **0.06** | **0.01** | **0.12** | *<0.0001* |
| **HexCer/Cholesterol** | **-0.11** | **-0.19** | **-0.04** | 0.08 | 0.00 | 0.17 | *<0.0001* |
| **LacCer d18:1** | -0.19 | -0.53 | 0.14 | 0.02 | -0.40 | 0.44 | *0.25* |
| **Total Cer** | -0.51 | -1.54 | 0.53 | **-1.39** | **-2.56** | **-0.22** | *0.02* |
| **Total Cer/Cholesterol** | -0.05 | -0.18 | 0.08 | -0.06 | -0.21 | 0.09 | *0.71* |
| *Aminoacids in µmol/L* |  |  |  |  |  |  |  |
| **Alanine** | 13.00 | -13.72 | 39.74 | **-51.27** | **-81.10** | **-21.44** | *<0.0001* |
| **Arginine** | **7.51** | **2.81** | **12.13** | **6.19** | **0.74** | **11.64** | *0.006* |
| **Asparagine** | **-18.85** | **-21.64** | **-16.08** | **-22.08** | **-25.45** | **-18.71** | *<0.0001* |
| **Aspartic acid** | **7.85** | **4.90** | **10.80** | 3.10 | -0.32 | 6.52 | *<0.0001* |
| **Citrulline** | -0.12 | -2.79 | 2.55 | 0.05 | -3.61 | 3.70 | *0.99* |
| **Glutamine** | 1.89 | -39.79 | 43.58 | -33.03 | -80.28 | 14.22 | *0.19* |
| **Glutamic acid** | **91.37** | **63.87** | **118.86** | **77.32** | **44.67** | **109.98** | *<0.0001* |
| **Glycine** | **52.58** | **26.63** | **78.53** | **67.45** | **37.01** | **97.90** | *<0.0001* |
| **Isoleucine** | **11.92** | **7.46** | **16.39** | **9.87** | **4.01** | **15.72** | *<0.0001* |
| **Leucine** | 1.57 | -5.97 | 9.11 | -4.03 | -13.36 | 5.29 | *0.30* |
| **Lysine** | -4.18 | -14.94 | 6.58 | **-26.46** | **-39.41** | **-13.50** | *<0.0001* |
| **Methionine** | **5.27** | **3.71** | **6.83** | **4.157** | **2.23** | **6.05** | *<0.0001* |
| **Ornithine** | 0.27 | -6.81 | 6.26 | **-9.85** | **-16.50** | **-3.19** | *0.006* |
| **Phenylalanine** | **5.44** | **2.17** | **8.71** | 1.68 | -2.14 | 5.50 | *0.002* |
| **Proline** | **-61.25** | **-82.30** | **-40.21** | **-71.95** | **-96.77** | **-47.14** | *<0.0001* |
| **Serine** | 5.56 | -1.71 | 12.83 | **9.73** | **1.40** | **18.06** | *0.07* |
| **Tryptophan** | 1.27 | -4.19 | 1.65 | **-3.67** | **-6.87** | **-0.47** | *0.04* |
| **Tyrosine** | **6.56** | **2.38** | **10.74** | **5.13** | **0.48** | **9.79** | *0.009* |
| **Valine** | **35.07** | **19.80** | **50.33** | **30.11** | **8.71** | **51.52** | *<0.0001* |
| *Acylcarnitines in µmol/L* |  |  |  |  |  |  |  |
| **C0** | -0.16 | -2.12 | 1.79 | -1.82 | -4.12 | 0.49 | *0.19* |
| **C2** | -0.13 | -0.67 | 0.41 | -0.51 | -1.10 | 0.08 | *0.18* |
| **C3 (*10^-2^)** | 3.16 | -0.84 | 7.16 | 1.27 | -3.81 | 6.34 | *0.26* |
| **C4 (*10^-2^)** | -1.42 | -6.43 | 3.59 | **-5.47** | **-10.18** | **-0.76** | *0.002* |
| **C5 (*10^-2^)** | **-1.27** | **-2.41** | **-0.12** | **-1.59** | **-2.88** | **-0.29** | *0.048* |
| **C6 (*10^-2^)** | 0.48 | -0.44 | 1.49 | 0.98 | -0.28 | 2.25 | *0.29* |
| **C8 (*10^-2^)** | -0.77 | -3.42 | 1.88 | 1.75 | -2.59 | 6.09 | *0.44* |
| **C10** | **-0.05** | **-0.09** | **-0.00** | 0.00 | -0.06 | 0.07 | *0.04* |
| **C12 (*10^-2^)** | -0.80 | -1.85 | 0.25 | -0.79 | -2.29 | 0.70 | *0.31* |
| **C14 (*10^-2^)** | **-0.92** | **-1.34** | **-0.50** | **-0.94** | **-1.45** | **-0.43** | *<0.0001* |
| **C16 (*10^-2^)** | **-1.75** | **-2.53** | **-0.98** | **-2.01** | **-2.90** | **-1.12** | *<0.0001* |
| **C18 (*10-2)** | **-1.16** | **-1.54** | **-0.78** | **-1.05** | **-1.45** | **-0.65** | *<0.0001* |
| **C10:1 (*10-2)** | **9.48** | **6.48** | **12.48** | **7.16** | **2.59** | **11.73** | *<0.0001* |
| **C12:1 (*10^-2^)** | -1.11 | -2.55 | 0.33 | -0.91 | -3.06 | 1.24 | *0.33* |
| **C14:1 (*10^-2^)** | -0.84 | -2.44 | 0.75 | -0.85 | -3.12 | 1.43 | *0.57* |
| **C14:2 (*10^-2^)** | **3.20** | **2.08** | **4.31** | **1.71** | **0.28** | **3.14** | *<0.0001* |
| **C16:1 (*10^-2^)** | **-0.52** | **-0.89** | **-0.15** | **-0.68** | **-1.18** | **-0.17** | *0.01* |
| **C18:1 (*10^-2^)** | **-1.79** | **-2.82** | **-0.76** | **-1.63** | **-2.95** | **-0.32** | *0.003* |
| **C18:2 (*10^-2^)** | **3.66** | **2.88** | **4.44** | **1.79** | **0.78** | **2.80** | *<0.0001* |

^a^Adjusted for age, baseline body mass index and healthy diet; CI= 95%-confidence interval; P-value= p-value for the adjusted comparison between ethnic groups (F test). Please note that no corrections for multiple testing were applied. Bold marking indicates values that are significantly different from the European Dutch.
