# Supplementary material for: Ethnic differences in metabolite signatures and type 2 diabetes: a nested case–control analysis among people of South Asian, African and European origin
Source: Nutr Diabetes. 2017 Dec 19;7(12):300. doi: 10.1038/s41387-017-0003-z (PMC5865542; doi:10.1038/s41387-017-0003-z)
Supplement: Supplementary file 4 — Supplement 4 [file 41387_2017_3_MOESM4_ESM.docx]

**Supplement 4. The adjusted association of sphingolipids, aminoacids and AC with the cumulative prevalence of type 2 diabetes**

|  | **Adjustment for BMI change**^a^ | | | | **Additional adjustment for healthy diet**^b^ | | | |
| --- | --- | --- | --- | --- | --- | --- | --- | --- |
|  | **OR** | **CI lower** | **CI upper** | ***P value*** | **OR** | **CI lower** | **CI upper** | ***P value*** |
| *Sphingolipids* |  |  |  |  |  |  |  |  |
| **Cer d16:1** | 1.79 | 1.24 | 2.58 | *0.002* | 2.01 | 1.41 | 2.87 | *<0.0001* |
| **Cer d18:1** | 2.31 | 1.46 | 3.66 | *0.0003* | 2.38 | 1.52 | 3.74 | *0.0001* |
| **Cer d18:2** | 1.80 | 1.22 | 2.67 | *0.003* | 1.80 | 1.23 | 2.63 | *0.002* |
| **Gb3d18:1** | 0.66 | 0.49 | 0.90 | *0.008* | 0.66 | 0.44 | 1.00 | *0.05* |
| **Gb4 d18:1** | 0.98 | 0.66 | 1.45 | *0.40* | 0.97 | 0.67 | 1.40 | *0.86* |
| **HexCer d18:1** | 1.11 | 0.81 | 1.51 | *0.51* | 1.03 | 0.73 | 1.47 | *0.85* |
| **HexCer/Total Cer** | 0.44 | 0.31 | 0.64 | *<0.0001* | 0.36 | 0.23 | 0.57 | *<0.0001* |
| **HexCer/Cholesterol** | 0.88 | 0.65 | 1.21 | *0.44* | 0.81 | 0.57 | 1.15 | *0.23* |
| **LacCer d18:1** | 1.10 | 0.77 | 1.58 | *0.59* | 1.34 | 0.90 | 2.00 | *0.15* |
| **Total Cer** | 2.29 | 1.49 | 3.53 | *0.0002* | 2.37 | 1.54 | 3.62 | *<0.0001* |
| **Total Cer/Cholesterol** | 2.57 | 1.78 | 3.72 | *<0.0001* | 2.79 | 1.94 | 4.00 | *<0.0001* |
| *Aminoacids* |  |  |  |  |  |  |  |  |
| **Alanine** | 1.64 | 1.13 | 2.40 | *0.009* | 1.45 | 1.01 | 2.08 | *0.04* |
| **Arginine** | 1.05 | 0.79 | 1.41 | *0.72* | 1.25 | 0.91 | 1.70 | *0.16* |
| **Asparagine** | 0.46 | 0.28 | 0.74 | *0.001* | 0.37 | 0.20 | 0.68 | *0.001* |
| **Aspartic acid** | 1.12 | 0.82 | 1.54 | *0.47* | 1.54 | 0.97 | 2.43 | *0.12* |
| **Citrulline** | 0.95 | 0.73 | 1.24 | *0.71* | 1.06 | 0.80 | 1.41 | *0.68* |
| **Glutamine** | 0.53 | 0.36 | 0.77 | *0.0007* | 0.43 | 0.30 | 0.62 | *<0.0001* |
| **Glutamic acid** | 0.76 | 0.55 | 1.05 | *0.41* | 0.85 | 0.60 | 1.19 | *0.34* |
| **Glycine** | 0.63 | 0.43 | 0.92 | *0.02* | 0.87 | 0.59 | 1.28 | *0.46* |
| **Isoleucine** | 2.68 | 1.50 | 4.78 | *0.0008* | 2.75 | 1.57 | 4.82 | *0.0004* |
| **Leucine** | 2.31 | 1.49 | 3.59 | *0.0002* | 2.17 | 1.46 | 3.23 | *0.0001* |
| **Lysine** | 1.37 | 1.00 | 1.88 | *0.05* | 1.23 | 0.83 | 1.80 | *0.30* |
| **Methionine** | 1.12 | 0.83 | 1.51 | *0.46* | 1.30 | 0.96 | 1.77 | *0.09* |
| **Ornithine** | 0.76 | 0.57 | 1.03 | *0.07* | 0.79 | 0.57 | 1.09 | *0.14* |
| **Phenylalanine** | 1.54 | 1.13 | 2.09 | *0.006* | 1.33 | 0.97 | 1.83 | *0.08* |
| **Proline** | 0.92 | 0.65 | 1.30 | *0.64* | 0.89 | 0.59 | 1.36 | *0.60* |
| **Serine** | 0.62 | 0.46 | 0.83 | *0.001* | 0.70 | 0.96 | 1.00 | *0.05* |
| **Tryptophan** | 0.90 | 0.68 | 1.19 | *0.44* | 1.16 | 0.87 | 1.54 | *0.31* |
| **Tyrosine** | 1.49 | 1.03 | 2.17 | *0.03* | 1.26 | 0.85 | 1.86 | *0.25* |
| **Valine** | 1.87 | 1.24 | 2.82 | *0.003* | 2.04 | 1.27 | 3.27 | *0.003* |
| *Acylcarnitines* |  |  |  |  |  |  |  |  |
| **C0** | 1.35 | 1.03 | 1.78 | *0.03* | 1.28 | 0.97 | 1.68 | *0.07* |
| **C2** | 1.38 | 1.00 | 1.90 | *0.05* | 1.27 | 0.94 | 1.72 | *0.12* |
| **C3** | 1.33 | 0.99 | 1.78 | *0.06* | 1.36 | 1.03 | 1.78 | *0.03* |
| **C4** | 1.87 | 1.39 | 2.53 | *<0.0001* | 1.85 | 1.35 | 2.53 | *<0.0001* |
| **C5** | 1.79 | 1.26 | 2.55 | *0.001* | 1.78 | 1.27 | 2.49 | *0.0007* |
| **C6** | 1.48 | 1.04 | 2.10 | *0.03* | 1.46 | 1.12 | 1.91 | *0.005* |
| **C8** | 1.29 | 0.96 | 1.75 | *0.09* | 1.35 | 1.07 | 1.71 | *0.01* |
| **C10** | 1.21 | 0.86 | 1.69 | *0.27* | 1.28 | 1.00 | 1.64 | *0.04* |
| **C12** | 1.20 | 0.82 | 1.75 | *0.35* | 1.28 | 0.96 | 1.71 | *0.09* |
| **C14** | 1.26 | 0.85 | 1.88 | *0.25* | 1.39 | 1.01 | 1.92 | *0.04* |
| **C16** | 1.06 | 0.72 | 1.55 | *0.78* | 1.02 | 0.71 | 1.47 | *0.90* |
| **C18** | 0.51 | 0.59 | 1.22 | *0.36* | 0.82 | 0.59 | 1.14 | *0.23* |
| **C10:1** | 1.36 | 1.01 | 1.83 | *0.04* | 1.41 | 1.11 | 1.80 | *0.006* |
| **C12:1** | 1.02 | 0.76 | 1.36 | *0.92* | 1.09 | 0.84 | 1.41 | *0.53* |
| **C14:1** | 1.07 | 0.77 | 1.48 | *0.68* | 1.18 | 0.89 | 1.56 | *0.24* |
| **C14:2** | 1.09 | 0.78 | 1.54 | *0.61* | 1.19 | 0.89 | 1.57 | *0.23* |
| **C16:1** | 1.00 | 0.74 | 1.37 | *0.98* | 1.04 | 0.79 | 1.37 | *0.80* |
| **C18:1** | 0.96 | 0.69 | 1.33 | *0.78* | 0.98 | 0.72 | 1.34 | *0.89* |
| **C18:2** | 0.99 | 0.75 | 1.30 | *0.93* | 1.09 | 0.84 | 1.43 | *0.52* |

^a^Adjusted for age, ethnicity, and change in BMI between baseline and follow-up (instead of baseline body mass index); ^b^Adjusted for age, ethnicity, baseline body mass index, healthy diet.; OR= Odds ratio per standard deviation increase; CI= 95-% confidence interval; P-value= p-value for the adjusted association with type 2 diabetes (Likelihood ratio test). Please note that no corrections for multiple testing were applied
