# Supplementary material for: Ethnic differences in metabolite signatures and type 2 diabetes: a nested case–control analysis among people of South Asian, African and European origin
Source: Nutr Diabetes. 2017 Dec 19;7(12):300. doi: 10.1038/s41387-017-0003-z (PMC5865542; doi:10.1038/s41387-017-0003-z)
Supplement: Supplementary file 5 — Supplement 5 [file 41387_2017_3_MOESM5_ESM.docx]

**Supplement 5. Ethnic differences in the adjusted association of sphingolipids, aminoacids and AC with type 2 diabetes**^a^

|  |  | **OR** | **CI lower** | **CI upper** | ***P value interaction*** |
| --- | --- | --- | --- | --- | --- |
| *Sphingolipids* |  |  |  |  |  |
| **Cer d16:1** | South Asian Surinamese | 1.52 | 0.86 | 2.71 | *0.04* |
|  | African Surinamese | 3.91 | 1.80 | 8.52 |  |
|  | Dutch | 1.30 | 0.86 | 1.95 |  |
| **Cer d18:1** | South Asian Surinamese | 1.82 | 0.73 | 4.51 | *0.02* |
|  | African Surinamese | 5.42 | 2.40 | 12.22 |  |
|  | Dutch | 1.44 | 0.99 | 2.11 |  |
| **LacCer d18:1** | South Asian Surinamese | 0.62 | 0.38 | 0.99 | *0.006* |
|  | African Surinamese | 2.21 | 1.17 | 4.18 |  |
|  | Dutch | 0.71 | 0.33 | 1.52 |  |
| **Total Cer** | South Asian Surinamese | 1.72 | 0.76 | 3.87 | *0.03* |
|  | African Surinamese | 4.80 | 2.30 | 10.02 |  |
|  | Dutch | 1.49 | 1.01 | 2.21 |  |
| **Total Cer/Cholesterol** | South Asian Surinamese | 2.55 | 1.30 | 5.01 | *0.008* |
|  | African Surinamese | 5.27 | 2.34 | 11.88 |  |
|  | Dutch | 1.37 | 0.89 | 2.10 |  |
| *Aminoacids* |  |  |  |  |  |
| **Asparagine** | South Asian Surinamese | 0.61 | 0.36 | 1.03 | *0.09* |
|  | African Surinamese | 0.29 | 0.09 | 0.97 |  |
|  | Dutch | 0.16 | 0.05 | 0.52 |  |
| **Citrulline** | South Asian Surinamese | 0.72 | 0.45 | 1.15 | *0.10* |
|  | African Surinamese | 1.23 | 0.83 | 1.81 |  |
|  | Dutch | 0.68 | 0.37 | 1.25 |  |
| **Glutamine** | South Asian Surinamese | 0.45 | 0.29 | 0.71 | *0.04* |
|  | African Surinamese | 0.24 | 0.11 | 0.54 |  |
|  | Dutch | 0.90 | 0.49 | 1.67 |  |
| **Glycine** | South Asian Surinamese | 1.24 | 0.74 | 2.06 | *0.09* |
|  | African Surinamese | 0.72 | 0.41 | 1.27 |  |
|  | Dutch | 0.46 | 0.23 | 0.97 |  |
| **Proline** | South Asian Surinamese | 0.60 | 0.39 | 0.92 | *0.002* |
|  | African Surinamese | 0.74 | 0.28 | 1.96 |  |
|  | Dutch | 1.69 | 1.10 | 2.61 |  |
| **Tryptophan** | South Asian Surinamese | 0.76 | 0.57 | 1.02 | *0.02* |
|  | African Surinamese | 2.03 | 1.11 | 3.72 |  |
|  | Dutch | 0.80 | 0.48 | 1.31 |  |
| *Acylcarnitines* |  |  |  |  |  |
| **C0** | South Asian Surinamese | 1.01 | 0.65 | 1.57 | *0.06* |
|  | African Surinamese | 1.71 | 1.15 | 2.54 |  |
|  | Dutch | 0.84 | 0.50 | 1.41 |  |
| **C2** | South Asian Surinamese | 0.82 | 0.57 | 1.19 | *0.03* |
|  | African Surinamese | 1.87 | 1.13 | 3.08 |  |
|  | Dutch | 1.24 | 0.63 | 2.44 |  |
| **C10** | South Asian Surinamese | 1.00 | 0.47 | 2.15 | *0.05* |
|  | African Surinamese | 1.35 | 1.01 | 1.80 |  |
|  | Dutch | 0.50 | 0.23 | 1.05 |  |
| **C14:2** | South Asian Surinamese | 0.93 | 0.58 | 1.50 | *0.08* |
|  | African Surinamese | 1.20 | 0.85 | 1.71 |  |
|  | Dutch | 2.86 | 1.26 | 6.51 |  |

^a^Adjusted for age, ethnicity, baseline body mass index. ^b^ Could not be estimated. OR= Odds ratio per standard deviation increase; CI= 95%-confidence interval; P-value interaction= p-value for the interaction term ethnicity*metabolite (likelihood ratio test). Only metabolites with an estimated P≤ 0.10 for the interaction are shown.
